# Incidence and predictors of mortality among HIV positive children on anti-retroviral therapy in the selected health facilities of West Wollega Zone, Western Ethiopia: Retrospective cohort study

**DOI:** 10.1371/journal.pone.0314112

**Published:** 2025-01-24

**Authors:** Gelane Gurmu, Emiru Merdassa, Gemechu Tiruneh, Keneni Efrem, Firezer Belay, Lalisa Mekonnen, Jira Wakoya Feyisa, Matiyos Lema, Adisu Tafari Shama, Markos Desalegn

**Affiliations:** 1 Gimbi General Hospital, Oromia Regional Health Bureau, Gimbi, Ethiopia; 2 Departments of Nursing, School of Nursing and Midwifery, Institute of Health Science, Wollega University, Ethiopia; 3 Departments of Public Health, Institute of Health Sciences, Wollega University, Ethiopia; Madda Walabu University, ETHIOPIA

## Abstract

**Introduction:**

The mortality rate among Human immunodeficiency Virus (HIV) who have started antiretroviral therapy (ART) continues to be increased in resource-limited countries, despite a decline in developed nations. Furthermore, research within this age group is limited and has not previously been conducted in the study area. Consequently, this study aimed to determine the incidence of mortality and its predictors among HIV-positive children who have been receiving ART at public health facilities in West Wollega.

**Objective:**

To assess incidence rate and predictors of mortality among HIV-positive children on ART at selected health facilities of West Wollega, Ethiopia, 2022.

**Methods:**

A retrospective cohort study design was conducted. A simple random sampling method was employed to select 286 children living with HIV who started ART from 01 January 2012 to 31 October 2021. Data were entered into Epi-Data Version 3.1, and STATA Version 14 was used for statistical analysis. A Kaplan-Meir survival curve and Long Rank test were used to estimate survival probability and assess statistical differences. The Cox regression model was used to determine independent predictors of mortality.

**Results:**

The total follow-up time was 15, 652 child-months, and the overall incidence of mortality was 1.92 (95%CI: 1.34, 2.74) per 1000 child-months. The median time to death following the initiation of ART was 6 months. This study also showed that children with WHO clinical stage III (AHR = 3.4, 95% CI: 1.2, 7.4), stage IV (AHR = 5.4, 95%CI: 1.5, 19.8), Being anemic (AHR = 4.9, 95%CI: 1.8, 13.4), CD4 cell count below threshold (AHR = 3.7, 95%CI = 1.4, 9.5), delayed developmental milestone (AHR = 4.5, 95%CI: 1.7, 11.7) were at higher risk of mortality.

**Conclusion:**

The overall mortality rate was lower compared to the previous study findings. Anemia, WHO clinical stage, CD4 cell count, and delayed developmental milestones were independent predictors of mortality. Therefore, the focus should be given to all children on ART during the early periods of ART initiation, advanced HIV disease, presence of anemia, severe immune deficiency, and delayed developmental milestones.

## Introduction

The human immune deficiency virus (HIV) disease progression is rapid in children and nearly half of them would be dying by the second year of infection if left untreated. This disease remains the major public health problem worldwide in which the low- and middle-income countries are particularly affected. The United Nations Program on HIV/Acquired immunodeficiency syndrome (UNAIDS) reported, 37.7 million people were living with HIV worldwide of whom 1.7million were children aged <15 years [1].

HIV affects children at all development stages. Children with HIV are more likely to be stunted, go through puberty later than their peers, neurocognitive delays associated with behavioral troubles and high proportion of mental illness [2]. The main contributing factors of mortality among HIV positive children were severe immune deficiency, low hemoglobin level, advanced WHO clinical stage, being young, poor ART adherence, under nutrition, presence of baseline opportunistic infection, and delayed developmental milestones [3–6].

The start of antiretroviral therapy (ART) has been shown to reduce HIV-related morbidity, mortality and risk of infection [7]. Early diagnosis increases the early detection of HIV-positive children. Early starting of antiretroviral treatment for those infected remains low [8, 9]. Globally, 27.5 million people living with HIV were receiving antiretroviral therapy and of which 54% of them were children [10]. Despite increased ART services in the world, in Africa, there is still high child death. Studies conducted in sub-Saharan African countries: South Africa, Nigeria, Kenya and Ethiopia showed a mortality rate of 4.7, 1, 8.4, and 12.58 deaths per 100 child years respectively [4, 11–14].

The scarcity of evidence is one of these gaps. The majority of previous studies were focused on the hospital only; however, this study included both hospital and health centers to increase generalizability. With the aim of further understanding the incidence and predictors of mortality is important step to identify possible intervention to improve the quality of care for HIV positive children and reduce mortality.

## Method and materials

### Study area and period

The study was conducted in the health facilities of West Wollega Zone. Ghimbi is the Capital city of West Wollega Zone located 441 km from Addis Ababa. West Wollega climatically Wayne Dega and the rainfall is about seven months from March to September in the year. It is bordered in the east-by-East Wollega, the south by Benishangul, in the North by Ilu Abba-Bor, and in the west by Kellem Wollega. The zone has 19 woreda and 2 administrative towns. The total population is estimated to be 2 million people [15]. There are 7 hospitals and 67 health centers that render preventive, curative, and rehabilitative services for the catchment area population. From these, 19 health centers and 6 hospitals are giving ART services. In this zone, 384 children were ever enrolled in ART during the study period. Patients enrolled in HIV care are followed based on national HIV treatment guideline and WHO recommendations. In this study, five hospitals (Gimbi Adventist Hospital, Gimbi General Hospital, Aira General Hospital, Nedjo Hospital, and Begi Hospital) and four health centers (Gimbi Health Center, Inango Health Center, Haru Health Center, and Nole Health Center) were selected using simple random sampling. The study period was between January 1, 2012, and October 30, 2021. Data extraction was conducted from patient charts from December 20, 2021, to January 30, 2022.

#### Study design

A retrospective cohort study design was employed.

#### Source population

All under fifteen children with HIV/AIDS and started ART Treatment in West Wollega zone health facilities.

#### Study population

The study population was all HIV-positive children under the age of 15 who initiated ART treatment at selected health facilities in West Wollega between January 1, 2012, and October 30, 2021. The study included HIV-positive children aged less than 15 years who started ART treatment were included, while excluding those with incomplete medical records, specifically those without recorded outcome, date admission and date outcome occurred. Children who transferred in without baseline data were also excluded.

#### Sample size determination and sampling procedure

Sample size was calculated using STATA version 14 command ***“stpowerlogrank 0*.*5*, *hratio () power (0*.*8) wdprob (0*.*15)”*,** taking HR for different variables, Log-rank 0.5, and withdrawal probability 15% for non-response rate. The hazard ratio was taken from a previous study conducted at Debre-Markos Referral Hospital for WHO clinical stage, ART adherence, CD4 Count, and OI prophylaxis [3]. Finally, the maximum sample size was obtained from OI prophylaxis, 298 HIV/AIDS positive children were selected for this study.

**Fig 1:**
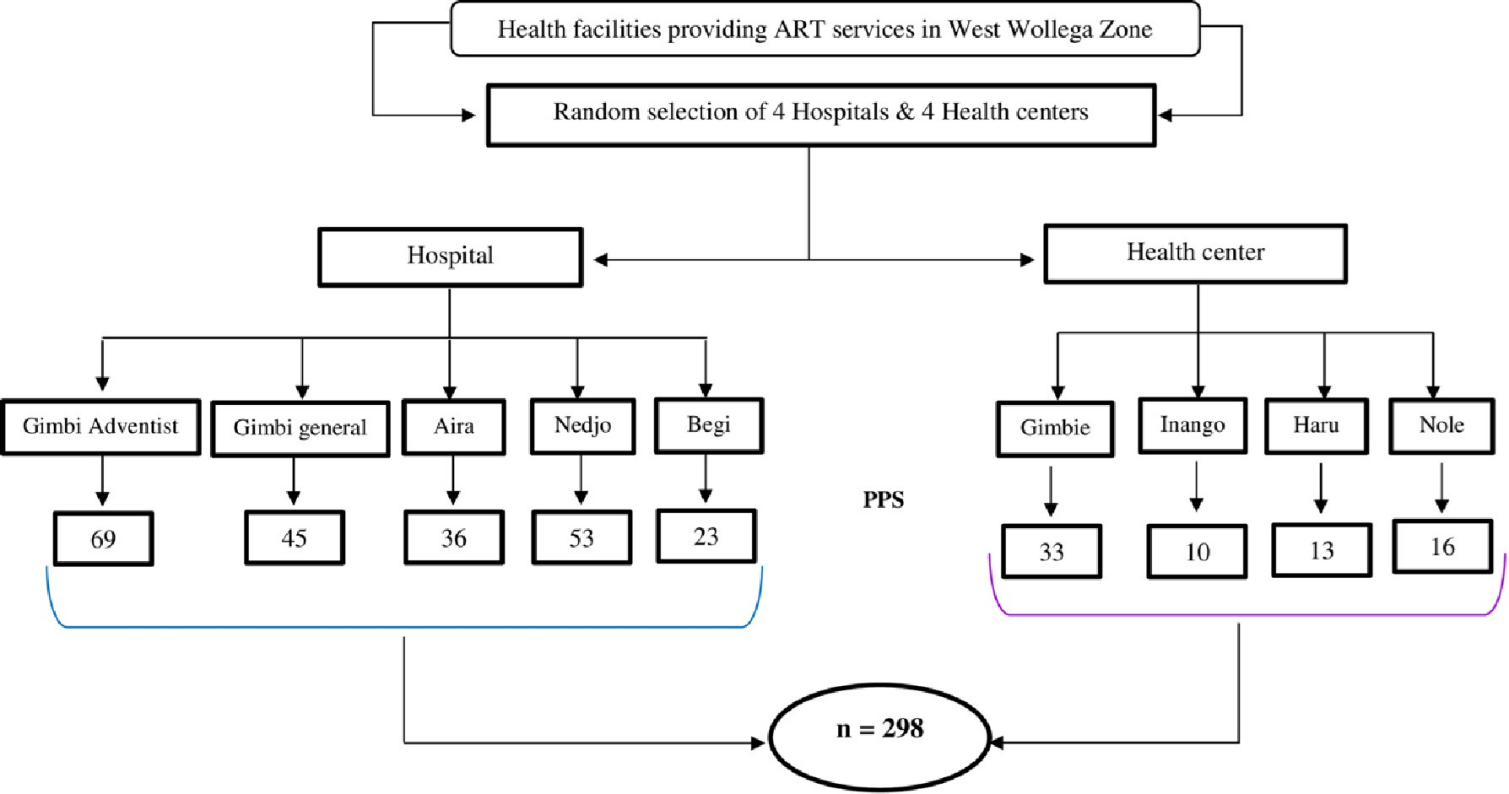
Schematic presentation of sampling procedure.

Data were collected from randomly selected health facilities (5 hospitals and 4 health centers). All under 15 children-initiated ART between January 1, 2012 and 31, 2021 in the studied hospitals and health centers were 384. The proportion was calculated as follows: calculated sample size (298) divided by the total number of HIV-infected children who started ART in the nine health facilities (five hospital & four health centers) between January 1, 2012 and October 31, 2021 multiplied by the total number of HIV-infected children on ART at a given health facility between January 1, 2012 and December 31, 2021(Fig 1). Finally using simple random sampling technique, 298 child cards were retrieved.

#### Variables measurements and operational definitions

The outcome variable in this study was the time to death, which was estimated in months and calculated from the time the children started ART treatment until event occurred. Independent variables were classified into; Socio-demographic variables included Age, sex, education level of caregiver, employment status of caregiver and residence; Clinical variables included-presence of opportunistic infections, types of opportunistic infection, presence of TB, presence of TB treatment, developmental milestones, WHO clinical stage, drug adherence, presence of cotrimoxazole prophylaxis, Height, weight, functional status, and drug side effect; Immunologic related variables included CD4 Count, viral load, immunization status and hemoglobin level.

**Good ART drug adherence**:—Children on ART who were taking or only missed 1dose out of 30 doses or two doses from the 60 doses imply.

**Fair ART drug adherence**:—Children missed 2–4 doses out of 30 doses or 4 to 9 doses out of 60 doses;

**Poor ART drug adherence**:—Children missed ≥5 doses out of 30 doses or ≥ 10 doses out of 60 doses implies poor adherence [16].

**Suppressed viral load**: It was a threshold of less than 1000 RNA copies/ml

**Unsuppressed viral load**: It was a threshold of greater than 1000 RNA copies/ml [8].

**Lost to follow up:—**If a patient discontinued ART for one month as recorded by the ART clinician [17]. Children considered as dropout if they discontinued ART for at least three months as recorded by the ART clinician [17].

**Children**:—This study considered under 15 to be children in line with WHO guidelines to make a comparison with previous study findings.

**Children’s CD4 cell count**: According to the World Health Organization’s age-appropriate classification, the immunologic levels of were measured and classified. For children under age of one, those with a CD4 cell count of less than 1500 cells/mm^3^; for children aged one to under three years, those with a CD4 cell count of less than 750 cells/mm^3^; for children aged three to under five, those with a CD4 cell count less than 350 cells/mm^3^ and children aged five to under fifteen those with a CD4 cell count less than 200 cells/mm^3^ were categorized as having a low immunologic level [18].

**Anemia**: Children were categorized as anemic if their hemoglobin levels were below the following thresholds, based on age. For children aged between 6 to 59 months, a hemoglobin less than11g/dl; for children between 5 to 12 years, a hemoglobin less than 11.5g/dl; and for children aged between12 to 15 years, a hemoglobin level of less than 12g/dl was the criterion anemia [19].

**Nutritional status** The nutritional status of under five children was measured and categorized based on standard deviation score of(-2SD) using WHO standard. For the children aged between 5 to 15 years, their body mass index (BMI) for age was measured, and those with a BMI for age less than -2SD were classified as undernourished [20].

**Censored:** The child has not experienced the event of death before the end of the study, before being lost to follow-up or transferred out.

**Event**: The child experienced the event, death during the follow-up period.

### Data extraction tools and procedure

The data source for this study was secondary data obtained from medical records of all children under 15 years of age who started ART between January 1, 2012, and October 30, 2021. The data collection form was adapted from the previous study [21]. To collect the necessary data, the ART registries were used including the intake form, Pre-ART register, ART register and follow up forms. The data was extracted by one ART data clerk working in the ART clinic from each health facility. In this study, the sampling frames were those who had been registered for chronic care during the data retrieval period of ten consecutive years from January 2012 to October 2021 in the health facilities of West Wallaga. Finally, All HIV-positive children on care and support follow-up who had started ART at health facilities of West Wallaga and fulfilled the inclusion criteria were selected.

### Data quality assurance

The prepared data extraction tool was pretested on 5% sample of the study population from hospital records of all children under15 years of age who initiated ART from the period of January 1, 2012, to October 30, 2021, at Gimbi General Hospital. Modifications to the content and simplification of the questionnaires were made following pre-testing. One data collector and one supervisor were assigned for each selected health facility. The data collectors and supervisors were trained for one day before the actual data collection period. Filled questionnaires were checked for completeness. Data cleaning was conducted at the end of data collection before analysis.

### Data management and analysis

Epi-Data Version 3.1 was used for data entry, while STATA Version 14 was used for statistical analysis. Prior to analysis, data was cleaned. Simple frequency table, cross-tabulation, re-categorization of categorical variables and categorization of continuous non-normal variables was done to be suitable for statistical analysis. The Kaplan Meier survival curve was used for the estimation of overall survival probability, and a Log-rank test was used to test for statistical differences. The month was used as a time scale to calculate the median time to death.

A Cox proportional hazards regression model was used to determine factors associated with mortality. Independent variables with P-value < 0.25 in the bi-variate Cox-regression model was considered potential candidates for inclusion included in multivariable Cox regression analysis. The proportional hazard assumption was tested by a Global test based on Schoenfeld residuals and by examining the log minus log plot. Backward stepwise procedures were used to retain the significant variables in the final model. The Log-likelihood (LL) value was considered to select the best-fit model. Model adequacy was assessed for the variables that remained in the Multivariable model using the Schoenfeld residuals test. Finally, the Cox Snell residual graph was used to assess overall model adequacy of the proportional hazard model. The final model was interpreted using an adjusted Hazard Ratio (AHR) with a 95% confidence interval and p-value < 0.05 to measure the risk of death between different levels of independent variables.

### Ethical consideration

Ethical approval was received from the Wollega University Institute of Health Sciences Research Ethical Review Committee. A formal letter of cooperation was written to selected respective hospitals and Health centers and permission was also obtained from the hospital administrator and Health center director. Data were collected after official permission was obtained. No personal identifiers were used on the data collection form.

## Results

### Socio-demographic characteristics of children

After reviewing the medical records of 298 HIV-infected children, 286 records were included in the final analysis, while 12 records were excluded due to unknown status and unrecorded time (date of ART drugs initiation and date of exit). The age of the children ranges from 1–14 years with a median age of 6-years (IQR = 2–14 years) at the time of ART initiation and nearly half (47.1%) of children had started treatment before their fifth birth day. Of the total patients included in the study, more than half 164 (57.3%) were male, and about three fourth of the children were from urban. The majority (86.4%) of the children live with their parents (Table 1).

**Table 1:** Socio-demographic characteristics of children started ART in health facilities of west Wollega zone, Ethiopia, 2022.

Variables (n = 286)	Category	Frequency	Percent
Age in years	5 or above	157	54.9%
	1–4	129	45.1%
Sex	Male	122	42.6%
	Female	164	57.4%
Residence	Urban	215	75.17%
	Rural	71	24.83%
Educational status of mothers/care givers	Illiterate	98	34.27%
	Primary	96	33.57%
	Secondary	54	18.88%
	Tertiary	38	13.29%
Care giver	Parents	247	86.36%
	Other relative	39	13.64%

### Baseline clinical and immunological characteristics of children

**Fig 2:**
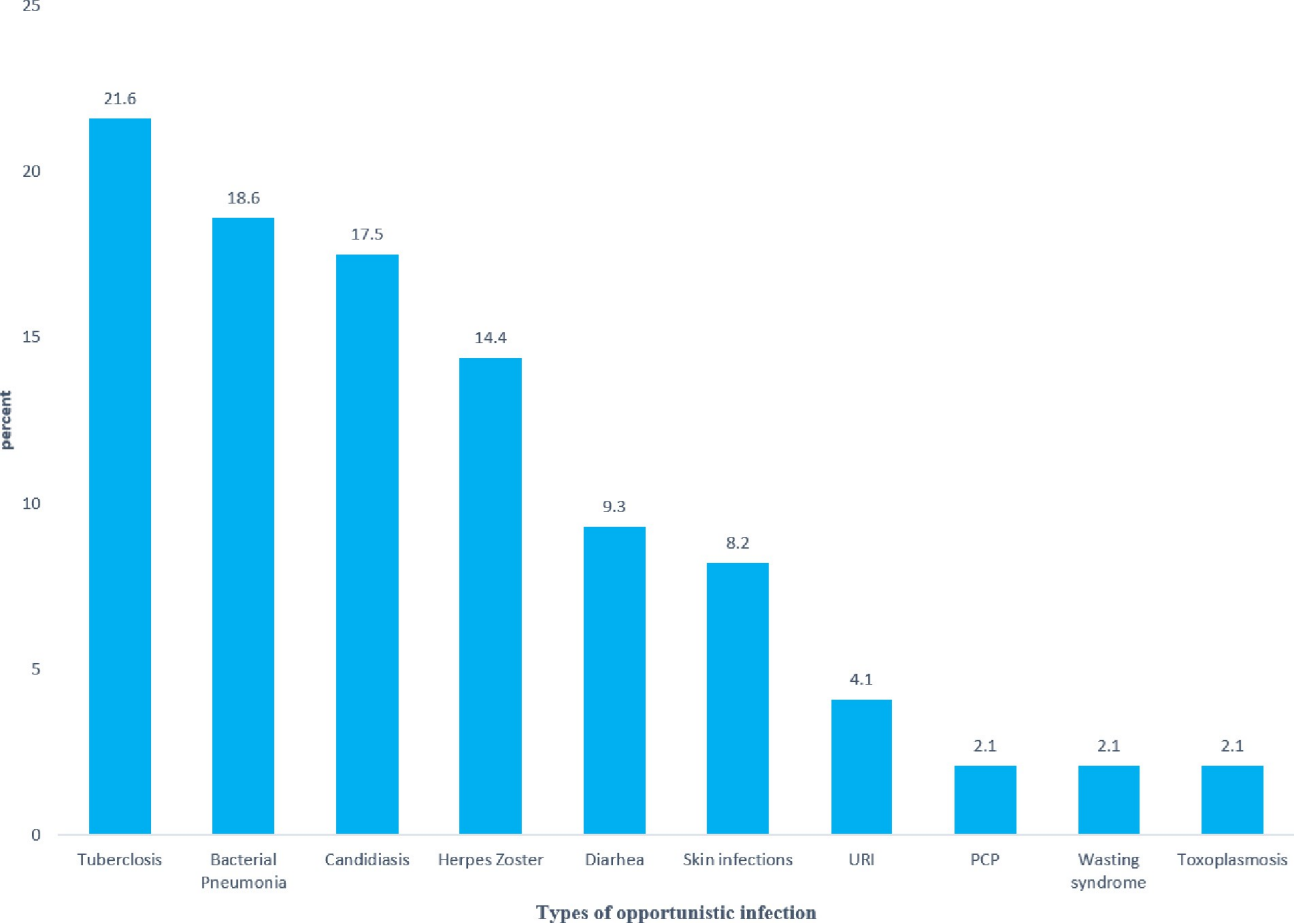
Percentage distribution of baseline opportunistic infections of HIV-positive children who started ART in the selected health facilities of West Wollega, Ethiopia, 2022.

According this study, 135 (47.2%) of children were in stage I&II, 89(31.1%) in stage III, and 62 (21.7%) were in stage IV of the WHO clinical stage at the start of ART. Regarding their CD4 cell count, 63 (24.6%) were below the threshold. The study also found that 6.67% of the children had anemia. Nearly half (48.6%) of them were undernourished, and 5.6% were at delayed developmental milestone status during ART initiation. Additionally, 7.69% of the children had a history of Tuberculosis at the start of ART. The data revealed that 78.7% of the children had good drug adherence, and 67.8% were given cotrimoxazole preventive therapy for prophylaxis of opportunistic infections (Table 2). Furthermore, 35.0% of the HIV-positive children included in the study had opportunistic infections (OIs) at ART initiation, with 21.4%, 18.6% and 15.4% of the children had tuberculosis, bacterial pneumonia and candidiasis respectively (Fig 2).

**Table 2:** Baseline clinical and immunological characteristic of children on ART at selected health facilities of West Wollega, 2022.

Variable n = 286	Category	Frequency	Percent
Cotrimoxazole use	Yes	194	67.8%
	No	92	32.2%
Developmental Milestone	Appropriate	268	93.7%
	Delayed	16	5.6%
	Regression	2	0.7%
Immunization status	Appropriate for age	224	78.3%
	Not appropriate for age	50	17.5%
	Not immunized	12	4.2%
TB Present	Yes	264	92.3%
	No	22	7.7%
TB Treatment	Received	264	92.3%
	Not received	22	7.7%
WHO Clinical Stage	Stages I&II	135	47.2%
	Stage III	89	31.1%
	Stage IV	62	21.7%
Hemoglobin Level (n = 255)	Non anemic	238	93.3%
	Anemic	17	6.7%
CD4 count	Above threshold	193	67.8%
	Below threshold	63	24.6%
Nutritional status	Normal	152	53.2%
	Undernourished	134	46.8%
Opportunistic infection present	Yes	89	34.6%
	No	187	65.4%

### Comparison of survival functions

**Fig 3:**
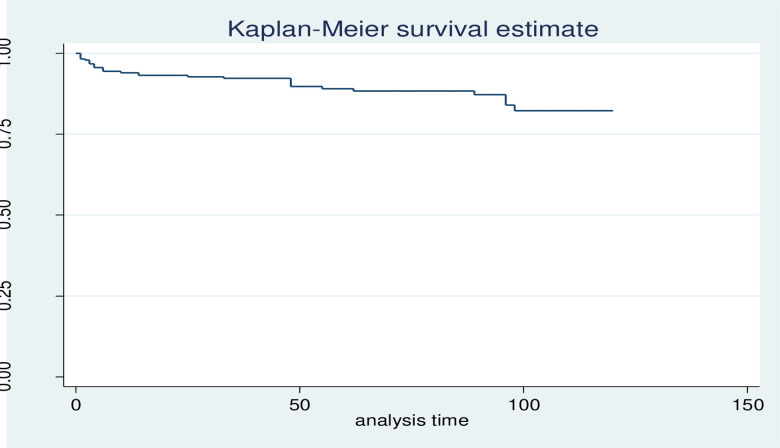
Kaplan-Meier curve of overall survival in HIV-positive children who started ART at selected health facilities of West Wollega zone.

The Kaplan Meier survival curve together with the log-rank test was used to check for the existence of any significant differences in survival probability between the various categories of variables considered in this study. Accordingly, the overall Kaplan-Meier survivor function estimate shows that most deaths occurred in the earlier months of ART initiation. Based on the findings, from the total of 30 deaths,12(40%) of them occurred in the first six months of follow-up and around 16(53.3%) deaths occurred in the first 12 months of follow-up, which declined through follow up time and continues steadily at later months of follow up (Fig 3).

**Fig 4:**
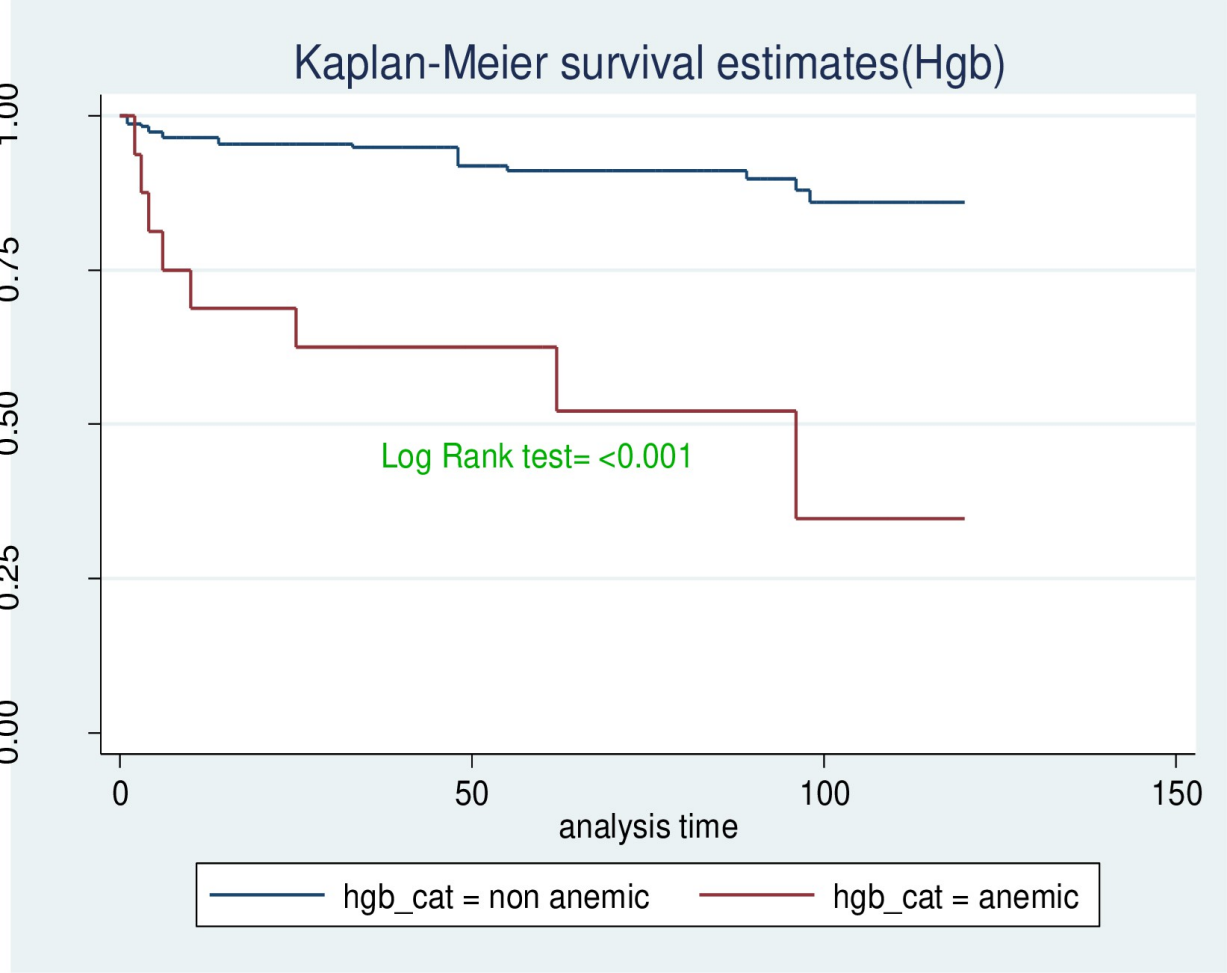
Kaplan-Meir survival curve by status of anemia, children who are on ART at selected health facilities in West Wollega zone, 2022.

**Fig 5:**
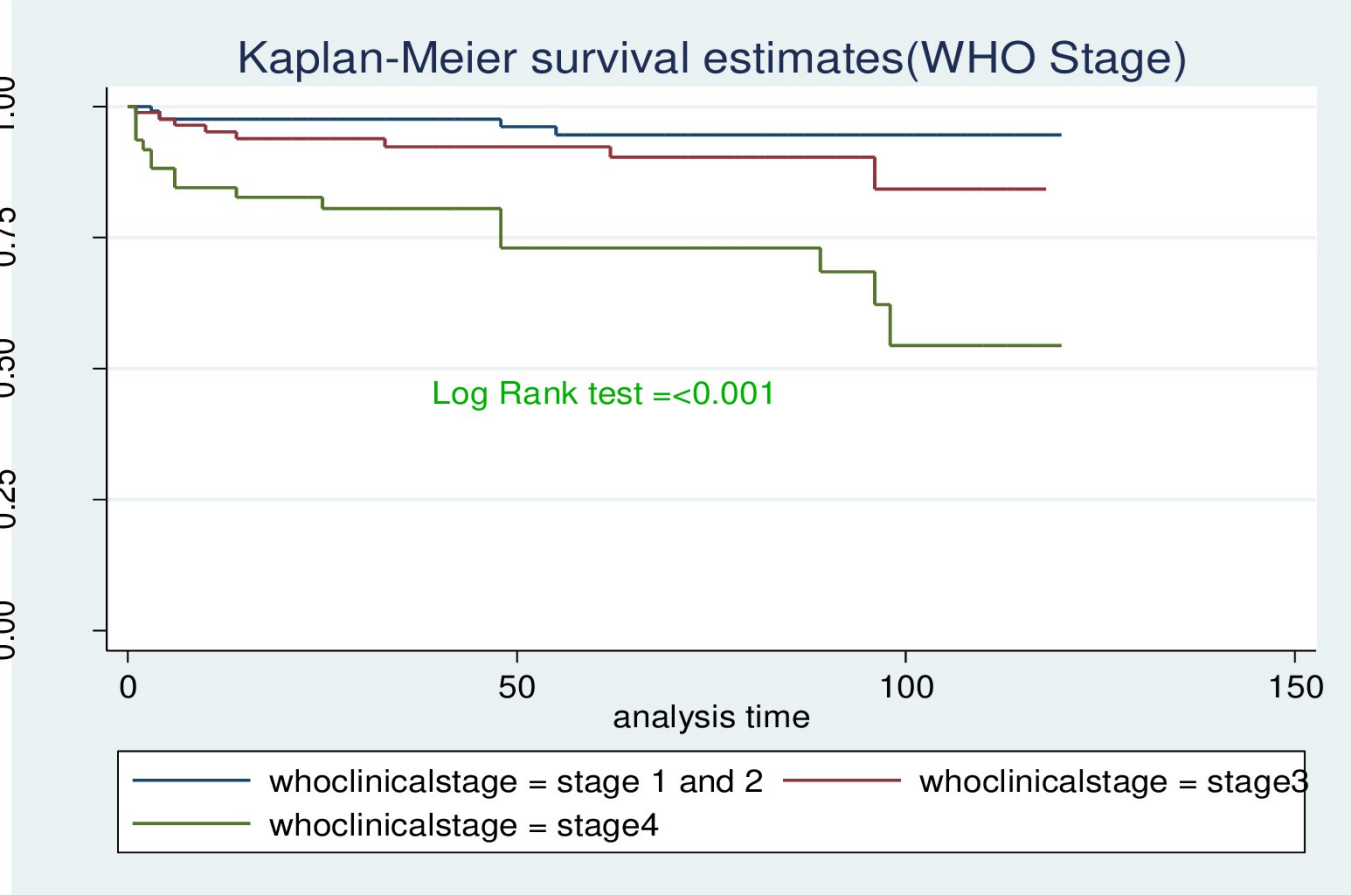
Kaplan-Meir survival curve by WHO clinical stages among HIV positive-children who are on ART at selected health facilities in West Wollega zone, 2022.

**Fig 6:**
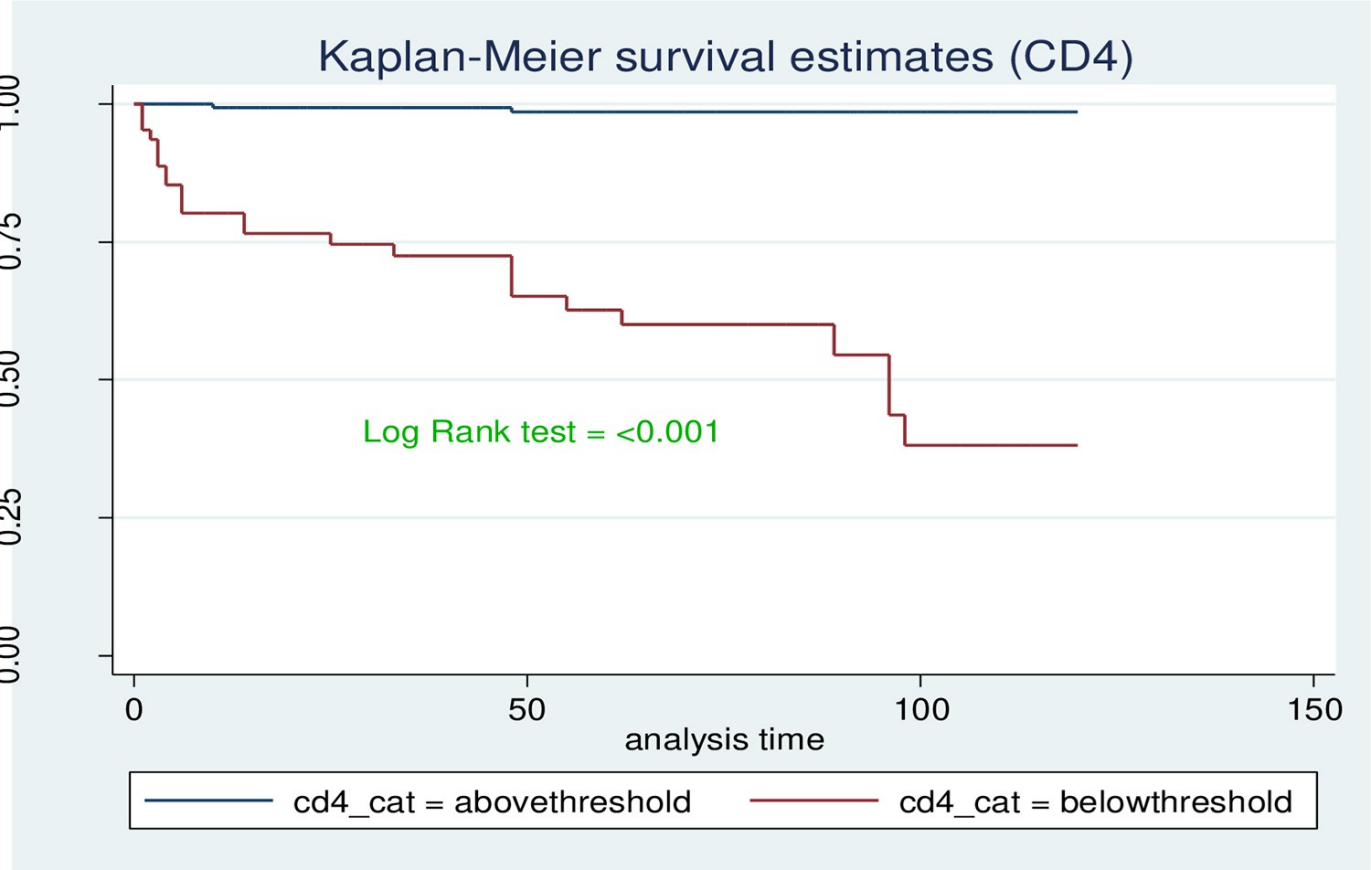
Kaplan-Meir survival curve by level of CD4 cell count among HIV positive children who are on ART at selected health facilities in West Wollega zone, 2022.

The survival probability for children with low hemoglobin level was significantly lower compared to those with normal hemoglobin level (log-rank, P<0.001). Children who began ART at the advanced stage of the disease (WHO stage III & IV) progression had significantly lower survival probability compared to those who started early in the disease progression (WHO clinical stages I & II). This visually observed difference was also statistically significant (Log-rank, p-value <0.001). As shown in Fig 5 below, the observed difference in shorter survival probability of children with CD4 cell count below the threshold compared with those children above the threshold was found to be statistically significant (Log-rank, p-value <0.001) (Figs 4–6).

### Incidence of mortality after initiation of ART

**Fig 7:**
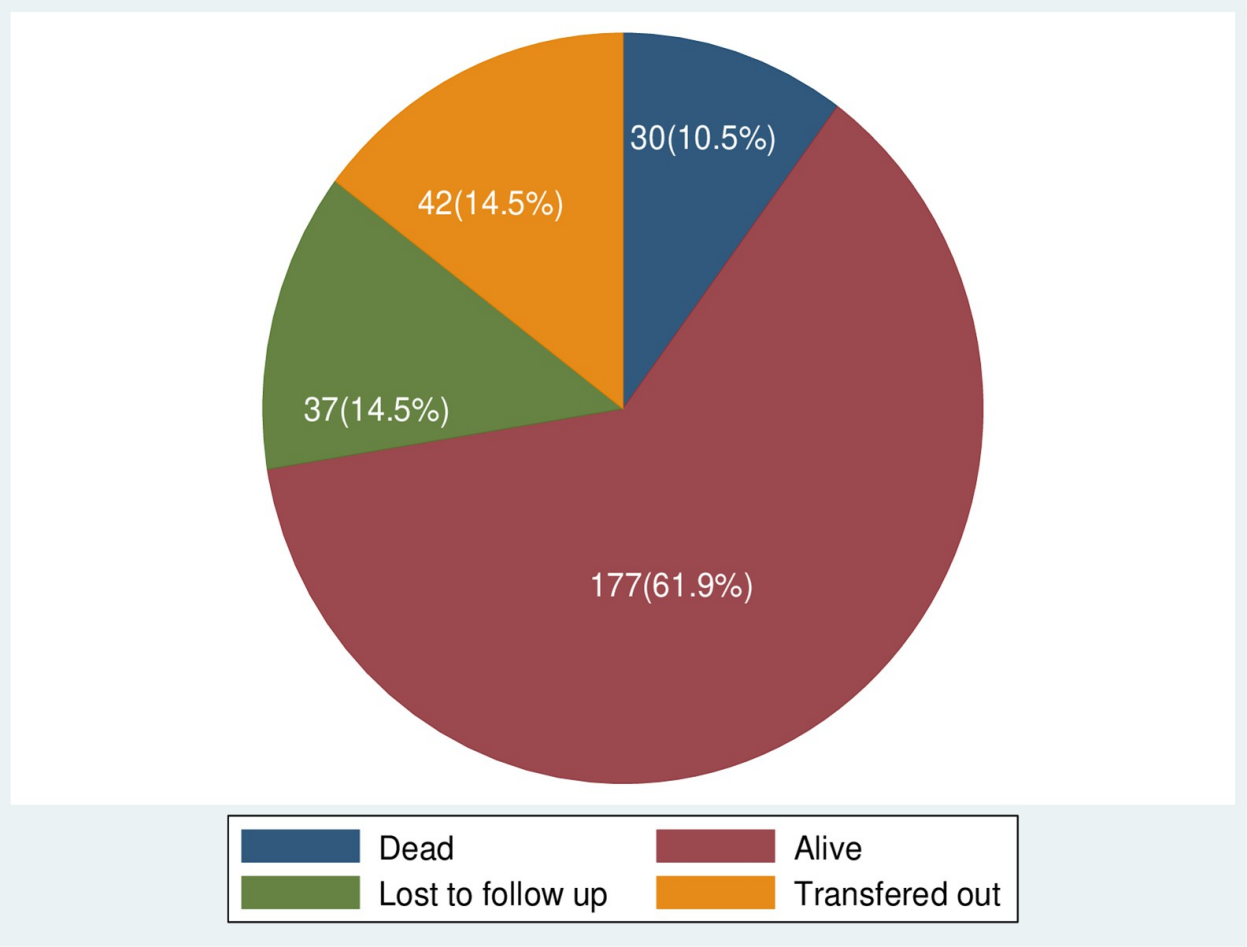
The outcomes of HIV positive-children who are on ART at selected health facilities in West Wollega, Ethiopia, 2022.

Out of the 286 cohort of children on ART, 177(61.89%) children were alive, 37(12.94%) were lost to follow-up, 42 (14.69%) were transferred out to other facilities and 30(10.49%) were reported dead (Fig 7). Of the 30 deaths, 12(40%) of them occurred within the first 6 months after initiation of ART. It is the highest proportion of death when compared to the subsequent time periods in the first year and second years after initiation of ART, which were respectively 16(53.3%), and 18(60%).

The cohort contributed to a total of 15652 person-months (1304.33 person-years) of follow-up with a median follow-up period of 49 months (IQR of 17 to 92 months). The overall mortality rate of the cohort was found to be 1.92 (95%CI: 1.34, 2.74) per 1000 children-months of observation or 2.3 (95% CI: 1.6, 3.28) per 100 Person- year of observation. The median time to death of children after initiation of ART was 6 months (IQR, 3.0–25.) The cumulative survival probability of children after 6, 12, 24, 36, 48, and 72 months were respectively 0.956, 0.931, 0.931, 0.922, 0.896, and 0.882 (Table 3).

**Table 3:** Life table cumulative survival of children after start of ART at a specific time, at selected Health facilities of west Wollega, Ethiopia, 2022.

Time in month	Number of children	Number of deaths	Survival probability	(95% CI:)	
0–6	286	12	0.9560	0.9238	0.9747
6–12	247	4	0.9401	0.9040	0.9629
12–18	230	2	0.9316	0.8935	0.9564
18–24	212	0	0.9316	0.8935	0.9564
24–30	200	1	0.9268	0.8875	0.9528
30–36	189	1	0.9218	0.8810	0.9490
36–42	176	0	0.9218	0.8810	0.9490
42–48	166	0	0.9218	0.8810	0.9490
48–54	148	4	0.8962	0.8474	0.9301
54–60	137	1	0.8895	0.8387	0.9250
60–66	127	1	0.8822	0.8293	0.9195
66–72	118	0	0.8822	0.8293	0.9195
72–78	111	0	0.8822	0.8293	0.9195
78–84	103	0	0.8822	0.8293	0.9195
84–90	90	1	0.8715	0.8137	0.9124
90–96	74	0	0.8715	0.8137	0.9124
96–102	55	3	0.8198	0.7328	0.8807
102–108	43	0	0.8198	0.7328	0.8807
108–114	30	0	0.8198	0.7328	0.8807
114–120	20	0	0.8198	0.7328	0.8807

#### Assessment of proportional hazards assumptions

To check whether the proportional hazard assumption was satisfied, the Schoenfeld residuals proportional hazard assumption test for the individual covariates, log-log plot, and global tests was used. Therefore, each covariate (P-Value > 0.05) and all of the covariates simultaneously (The global test for Cox proportional hazard was P-Value = 0.7735 > 0.05). The log-log plot graph and Global test of proportional hazard results showed that the proportional hazard assumption is satisfied (Table 4).

**Table 4:** Schoenfeld residuals test for proportionality assumption of each covariate and overall model of Cox proportional hazard.

Variable	Rho	Ch2	Df	P.value
WHO clinical stage	0.00614	0.00	1	0.9729
Presence of anemia	-0.07214	0.20	1	0.6562
CD4below threshold	0.08215	0.21	1	0.6464
Delayed developmental mile stone	0.00085	0.00	1	0.9965
Drug adherence	-0.06831	1.13	1	0.7205
Nutritional status	-0.21043	1.46	1	0.2275
Global test	-0.21043	5.66	9	0.7735

NB. *Rho*: *correlation coefficient between the residuals and time*

#### Predictors of child mortality after ART initiation

To identify potential independent variables for consideration in the multivariable Cox regression model, a bivariable analysis was performed for each covariate. Independent variable with p-value ≤ 0.25 in bivariable Cox regression analysis was selected for multivariable Cox regression. As a result, six variables were found to be a candidate for multivariable Cox regression model as shown in Table 5 below. These variables were ART drug adherence, delayed developmental milestone, WHO Clinical stages, presence of Anemia, CD4 Cell Count, and under nutrition. Finally, the six variables were fit to the multivariable cox regression model.

**Table 5:** Multivariable cox regression analysis for children who started ART at selected health facilities of west Wollega zone, Ethiopia, 2022.

Variables	Category	Survival status		CHR (95%CI)	AHR (95%CI)	P- value
		Death	Censored			
WHO clinical stages: at ART initiation	Stages I&II	5	130	1	1	
	Stage III	8	81	2.4(1.79, 7.4)	3.3 (1.2, 7.4)	0.002
	Stage IV	17	45	8.1 (2.9, 22.0)	5.4 (1.5, 19.8)	0.010
Hemoglobin level (in g/dl)	Non anemic	19	219	1	1	
	Anemic	8	9	6.7 (2.9, 15.3)	4.9 (1.8, 13.4)	0.002
Nutritional status:	Normal	19	133	1		
	Under Nutrition	11	123	0.58 (0.27, 1.22)	0.76 (0.32, 1.84)	0.545
CD4 Count	Above threshold	7	191	1	1	
	Below threshold	21	38	6.0 (3.9, 11.5)	3.7 (1.4, 9.5)	0.006
Developmental milestones	Appropriate	19	249	1	1	
	Delayed	11	7	13.5 (6.0, 30.7)	4.5(1.7, 11.7)	0.002
Drug adherence	Good	10	212	1	1	
	Fair	7	10	3.4(0.78, 15.4)	0.93(0.10, 8.00)	0.942
	Poor	13	34	8.1 (3.81, 17.4)	2.6(0.89, 8.14)	0.079

The backward stepwise selection was used and finally, the model with highest number of LLR was selected. Finally, only four predictors remained in the model. These 4 predictors were WHO Clinical stage, Anemia, CD4 Cell Count and delayed developmental milestone were found to have statistically significant association with mortality.

The adjusted hazard ratio for other covariates was presented in Table 5 below. It was observed in the final model that, the risk of death in children with WHO clinical stages III and IV were (AHR = 3.3, 95%CI: 1.2, 7.4, p-value = 0.002) and (AHR = 5.4, 95% CI: 1.5, 19.8, p-value = 0.010) respectively. Additionally, the hazard rate in children with low Hb level (AHR = 4.9, 95%CI: 1.8, 13.4, p-value = 0.002) almost 5 times higher than those who had normal Hb level. Furthermore, children who had CD4 cell count below threshold (AHR = 3.7, CI: 1.4, 9.4, p-value = 0.006) were 4 times more likely to die compared with those children above the threshold. Lastly, children with delayed developmental milestone at initiation of ART were almost 5 times more likely (AHR = 4.57, 95% CI: 1.78, 11.72, P = 0.002) to die early as compared to children with appropriate developmental milestone (Table 5).

### Model goodness-of-fit

**Fig 8:**
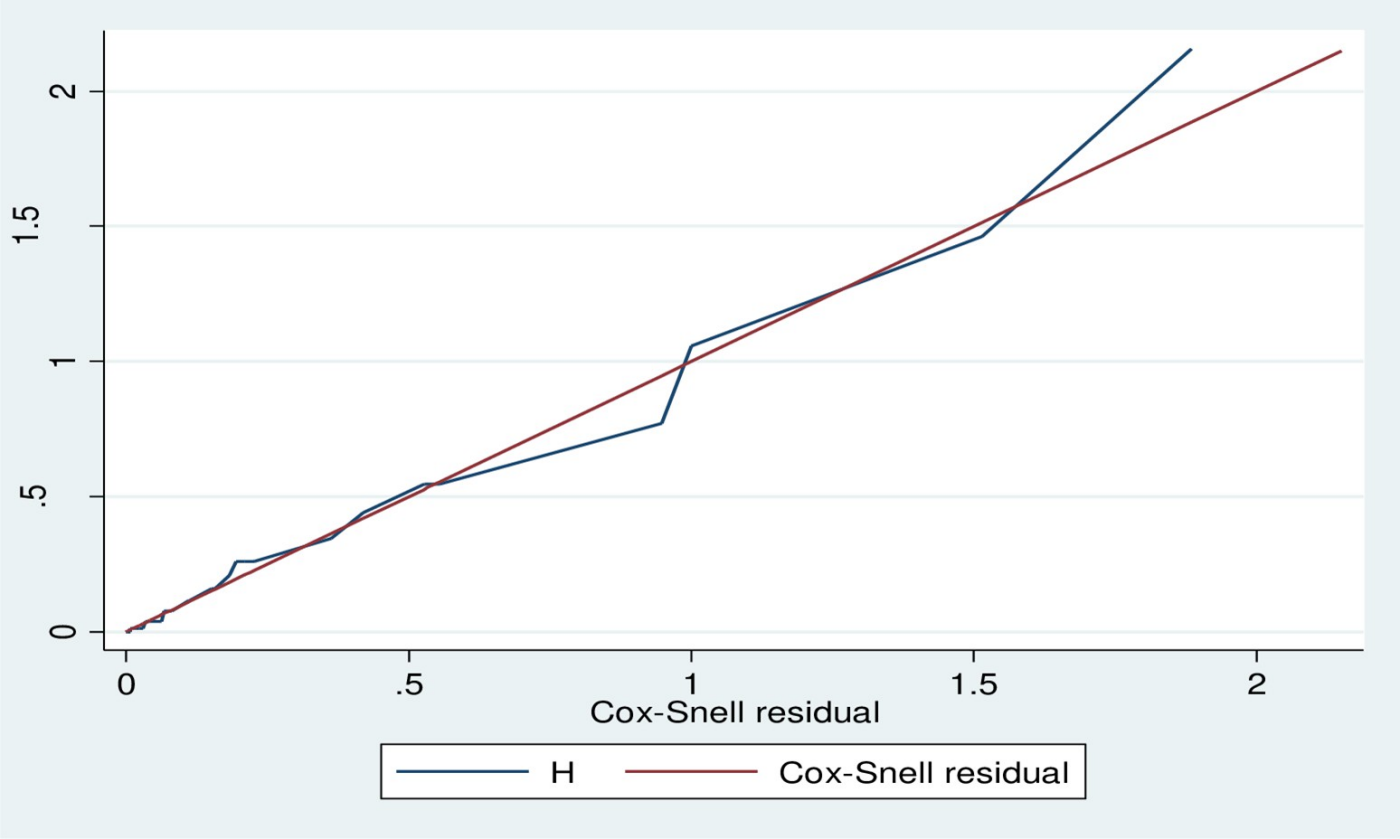
Cox Snell residual plot for overall adequacy of the model.

After fitting the multivariable Cox Proportional Hazard Model, overall model adequacy was assessed by using cox Snell residuals graph. Comparing the jagged line with the reference line (45 degree), we confirm that the Cox model approximately fit the data (Fig 8).

## Discussion

This study assessed the incidence and predictors of mortality among HIV positive children receiving ART in health facilities within the West Wollega Zone. A total of 286 children were followed for 15, 652 person-months of observation. During the follow-up period 30 (10.5%) died resulting in an overall incidence rate of 1.92 (95%CI = 1.34–2.74) per 1000 person-months of observation. This finding is comparable to the study findings reported from Adama referral hospital (2.06 deaths per 100 child-years) [12]. However, it is lower than the incidence rates reported in Addis Ababa Public Hospitals, Bahirdar Felegehiwot, Mekelle, Debre Tabor General Hospital and Dessie Referral Hospital, and Kenya [7, 13, 21–23]. The low mortality rate observed in this study could be attributed to various factors. Firstly, the disparities in all reported studies could be attributable to variations in sample size, follow-up period, and characteristics of research participants. Second, it is a long-term consequence, as the government is actively working to reduce the AIDS-related deaths by adopting different WHO ART standards.

According to this study, the cumulative survival probability of children after 3, 6, 12, 24, 36, 48, and 72 months was y 0.978, 0.956, 0.931, 0.931, 0.922, 0.896, and 0.882 respectively. Compared to studies conducted in Bahirdar and Adama, this study showed a higher probability of survival after ART initiation [12, 22].

Another finding obtained by this study was that from the total 30 deaths, the majority of them, 12(40%), occurred within the first 6 months after initiation of ART when compared to the proportion of deaths that occurred in the subsequent periods. The finding of this study coincides with the findings of the previous studies. A study conducted in South Africa reported 64% of deaths occurred within 3 months of ART initiation [14]. Similarly, in Nigeria, 81.3% and 84.4% of deaths occurred in the first 6 months and within 1 year respectively [24]. The study conducted in Adama and Zewuditu Memorial hospitals reported the majority of death within the first sixth months of starting ART [12]. The study conducted in Mekelle showed a high mortality in the first 12 months after ART initiation [25]. The elevated mortality rate during this period may have been attributed to the delayed use of ART services. The study revealed that over half the children were classified as WHO clinical stage III/IV, more than one-fourth of the children had a CD4 count below the threshold, and, more than half exhibited growth failure (underweight for age) at baseline.

This study identified the baseline WHO clinical stage as a significant predictor of mortality in HIV-positive children receiving ART. This study indicated that the risk of death was significantly higher in children with advanced WHO clinical stages (III and IV) compared to those in earlier stages (I and II). These results are consistent with previous studies conducted in Mekele, Bahirdar, Adama hospital, and Addis Ababa [12, 21, 22, 25]. It is anticipated that the children in advanced WHO clinical stages at the initiation of ART will have limited capacity for immune recovery, leading to an increased risk of life threatening opportunistic infections and early death.

Furthermore, the increased mortality risk in children with low hemoglobin (Hb) levels compared to children with normal hemoglobin levels. This finding aligns with the studies conducted in Zewuditu, Bahirdar Felegehiwot, Mekele, Adama hospitals and Kenya [13, 20, 22, 25]. Children presented with low hemoglobin levels at baseline necessitate early diagnosis and treatment of anemia, akin to interventions of HIV management [26]. Notably, ART drugs can directly induce anemia, particularly in children who already have anemia at baseline. Anemia may also signify pre-existing nutritional deficiencies, which are significant contributors of child mortality in developed countries [27, 28].

This study identified CD4 cell count as a significant predictor of mortality. This finding is consistent with studies conducted in Zewuditu, Bahirdar Felegehiwot, Mekele, Addis Ababa, Adama hospitals[21, 22, 29, 30]. Children with low CD4 count at baseline were four times more likely to die compared to those with higher CD4 cell counts. Children with low baseline CD4 counts were susceptible to varies diseases and infections that could lead to death. In Ethiopia, infectious diseases are the leading cause of death in children. These diseases are more severe and deadly in immunocompromised, including those with HIV. Even normal flora of the body can cause serious infection and lead to the death of HIV-positive children. ART cannot cure an existing diseases unless they have been treated.

Children who exhibited developmental delays at baseline had significantly higher risk of mortality compared to those who met expected developmental milestones. This finding was corroborated by studies conducted in North West Ethiopia, Bahirdar and a study conducted in Arba Minch town [31, 32]. Delayed developmental milestones at the early ART initiation may interfere with immune function and clinical recovery.

This study took into account an adequate duration of follow-up time and variety of healthcare facilities, including hospitals and health centers. However, this study relied on recorded data, which did not provide access to important independent variables for assessment.

## Conclusions

According to this study, a lower mortality rate was observed among the cohort of HIV-positive children receiving ART compared to the previous findings. However, the majority of deaths occurred within the first 6 months of ART initiation. WHO clinical stages, Anemia, CD4 cell counts, and developmental milestones were significant predictors of mortality.

Abbreviation and Acronyms AIDS:- Acquired Immunodeficiency Virus, AHD:- Adjusted Hazard Ratio, ART:- Antiretroviral Therapy, CHR:- Crude Hazard Rate, FMOH:- Federal Ministry of Health, HAART:- Highly Active Antiretroviral Treatment, HAPCOHIV/AIDS:- Prevention and Control Office, Hb:- Hemoglobin, HIV:- Human Immunodeficiency Virus, IQR:- Inter Quartile Range, LTFU:- Lost to Follow Up, MCV:- Measles containing vaccine, NA:- Not Applicable, PLHIV:-People Living with HIV,RHB:-Regional Health Bureau, TB:- Tuberculosis, UNAIDS: United Nations Program on HIV/AIDS, WHO:- World Health Organization, WU:-Wollega University.

## Supporting information

S1 Dataset (CSV)
